# Research Trends in Supercritical Fluid Extraction of Bioactive Compounds: A 30-Year Bibliometric Study from 1995 to 2025

**DOI:** 10.3390/molecules31142511

**Published:** 2026-07-18

**Authors:** Miguel A. Varas Condori, Luis Puente-Díaz, Adriano Costa de Camargo, Maritza Barriga-Sánchez

**Affiliations:** 1 Laboratorio de Compuestos Bioactivos, Centro de Innovación Productiva y Transferencia Tecnológica Pesquero, Acuícola, Agroindustrial Callao, Instituto Tecnológico de la Producción, Callao 07046, Peru; mbarriga@itp.gob.pe; 2 Departamento de Ciencias de los Alimentos y Tecnología Química, Facultad de Ciencias Químicas y Farmacéuticas, Universidad de Chile, Santiago 8380494, Chile; lpuente@ciq.uchile.cl; 3 Instituto de Ciencias Aplicadas, Universidad Autónoma de Chile, Santiago 7500910, Chile

**Keywords:** supercritical CO_2_, sustainable technologies, green extraction, Bibliometrix, sustainable development goals

## Abstract

The extraction of bioactive compounds using supercritical fluids has attracted increasing scientific attention as a sustainable alternative to conventional extraction methods. This study presents a bibliometric analysis aimed at providing an overview of the scientific development, collaboration patterns, and thematic trends in this research field. Bibliographic data were retrieved from the Web of Science Core Collection database for the period 1995–2025, resulting in a dataset of 2159 publications. The records were analyzed using the Bibliometrix package in R and complementary Web of Science tools to evaluate scientific productivity, leading contributors, collaboration patterns, keyword analysis (frequency and co-occurrence), thematic evolution, citation topics, and alignment with the United Nations Sustainable Development Goals. The results reveal sustained growth in scientific production, particularly during the last decade, reflecting the increasing interest in environmentally friendly extraction technologies and the growing demand for natural bioactive compounds. Brazil emerged as the leading contributor, followed by China and Spain. Collaboration patterns indicate the presence of international research partnerships among the most productive countries. Keyword analysis highlights antioxidant activity and polyphenols as the most frequent research topics. Thematic analysis reveals a progressive shift from compound characterization to process optimization and application-oriented research, with increasing emphasis on extraction performance, assisted extraction technologies, and the valorization of agro-industrial by-products as alternative sources of bioactive compounds. These findings provide a comprehensive overview of the evolution and main research directions in this field.

## 1. Introduction

The search for sustainable technologies for the extraction of bioactive compounds has gained increasing attention in recent decades, driven by the need to develop more efficient, safer, and environmentally friendly processes [1,2,3]. Bioactive compounds, such as polyphenols, carotenoids, and terpenoids, have attracted considerable interest due to their beneficial effects on human health and their potential applications in the food, pharmaceutical, and nutraceutical industries [4,5,6]. However, their extraction from plant matrices remains challenging due to the complexity of biological systems and the limitations associated with conventional extraction methods, which often involve the use of organic solvents, high temperatures, and long processing times [7]. These conditions may increase the environmental impact of the process as a result of high solvent consumption and, in some cases, degradation of thermolabile compounds [8].

In this context, the development of green extraction technologies has gained particular relevance as a strategy to improve process efficiency and reduce environmental impact [9]. Among these technologies, supercritical fluid extraction has been widely recognized as a sustainable alternative to conventional solvent-based methods, as it allows greater selectivity and efficiency in the recovery of bioactive compounds [10,11]. The foundations of supercritical fluid technology can be traced back to the studies conducted by Hannay and Hogarth in the late nineteenth century, who demonstrated the ability of fluids in the supercritical state to dissolve different solutes [12]. However, its industrial application began to develop in the 1970s with the use of supercritical carbon dioxide for coffee decaffeination, a process that led to numerous patents from 1974 onwards and the establishment of industrial plants capable of processing large product volumes [13,14]. Subsequently, during the 1980s, this technology began to be applied to the extraction of aromatic compounds, such as those present in hops, thereby expanding its use within the food industry [15]. Over the past decades, the use of supercritical fluids has been widely applied for the extraction of bioactive compounds from plant matrices, foods, and agro-industrial by-products [11,16].

Supercritical carbon dioxide (scCO_2_) is the most widely used solvent in these processes due to its favorable properties, including low critical temperature and pressure, non-toxic and non-flammable nature, low cost, and the ease with which it can be removed from the final extract through simple depressurization [17]. Although other supercritical fluids have also been investigated, their application is often limited by more demanding critical conditions. For example, water requires significantly higher temperatures and pressures (374.1 °C and 22.1 MPa), while polar solvents such as methanol (240.0 °C, 7.95 MPa), ethanol (243.1 °C, 6.39 MPa), and isopropanol (235.6 °C, 5.37 MPa) exhibit high critical temperatures [18].

In its supercritical state, CO_2_ exhibits physicochemical properties intermediate between those of a gas and a liquid, combining a liquid-like density that provides high solvation capacity with the low viscosity and high diffusivity typical of gases, facilitating penetration into the pores of solid matrices [19]. Moreover, slight variations in pressure or temperature allow the solvent power and selectivity of the fluid to be adjusted, enabling the recovery of bioactive compounds with different polarities [10]. These characteristics may improve extraction efficiency, reduce or eliminate the use of organic solvents, and allow operation at relatively low temperatures (35 to 60 °C), preserving thermolabile compounds [20] and producing high-purity extracts. Consequently, this technology has been recognized as a process aligned with the principles of green chemistry [10,21].

From a mechanistic perspective, supercritical fluid extraction involves solvent penetration into the matrix, dissolution of extractable compounds, and their subsequent transport to the separator [11]. The process is governed by both thermodynamic data (solubility and selectivity) and kinetic data (mass transfer coefficients). Typically, the extraction follows three stages: a constant extraction rate period dominated by convection, a falling extraction rate period where convection and diffusion coexist, and a diffusion-controlled period characterized by internal mass transfer limitations [22].

Several bioactive compounds correspond to secondary metabolites present in plants that, although not considered essential nutrients, can exert beneficial effects on human health, including antioxidant, anti-inflammatory, antimicrobial, and cardioprotective activities [4,5]. Among the main groups of bioactive compounds are polyphenols, carotenoids, terpenes and terpenoids, and alkaloids, whose structural diversity determines a wide range of biological properties and technological applications across different industrial sectors [23].

From a chemical perspective, these compounds exhibit different degrees of polarity, which significantly influence their behavior during extraction processes [24]. For example, carotenoids are characterized by their lipophilic nature and low polarity, whereas many phenolic compounds, such as flavonoids, exhibit higher polarity [25]. Because supercritical carbon dioxide is a relatively non-polar solvent, it is particularly efficient for extracting lipophilic compounds or those with low to intermediate polarity. In the case of more polar compounds, co-solvents such as ethanol are commonly used to modify the polarity of the medium and improve extraction efficiency [7,26,27].

In recent years, there has been growing interest in the valorization of agro-food by-products as alternative and sustainable sources of bioactive compounds [28]. By-products such as peels, seeds, leaves, and pomace from fruits and vegetables may contain significant concentrations of phenolic compounds, carotenoids, and other valuable bioactive compounds [29,30,31]. The utilization of these by-products can contribute to reducing the environmental impacts associated with their improper disposal, since large amounts of agro-food by-products such as fruit pomace are frequently discarded in landfills or left untreated despite their valuable bioactive composition [32]. Furthermore, their valorization supports more efficient resource management and promotes sustainable production models aligned with the Sustainable Development Goals (SDGs) established by the United Nations in the 2030 Agenda, particularly SDG 12 (Responsible Consumption and Production), as well as SDGs related to energy efficiency and sustainable industrial processes [33].

In recent decades, research on the extraction of bioactive compounds using supercritical fluids has increased considerably. In this context, bibliometric studies have become useful tools for analyzing the evolution of scientific production and identifying research trends. Some studies have applied this approach to the analysis of supercritical fluid research in specific areas, such as essential oil extraction or the synthesis of nanomaterials [34,35]. Therefore, the objective of this study was to perform a bibliometric analysis of the scientific research on the extraction of bioactive compounds using supercritical fluids, based on information obtained from the Web of Science database. The aim was to evaluate the evolution of scientific production, identify the most influential countries, authors, and institutions, analyze patterns of international collaboration, and explore the main thematic trends that have characterized the development of this research field.

## 2. Results and Discussion

### 2.1. Scientific Production and Productivity

#### 2.1.1. Annual Scientific Production

**Figure 1:**
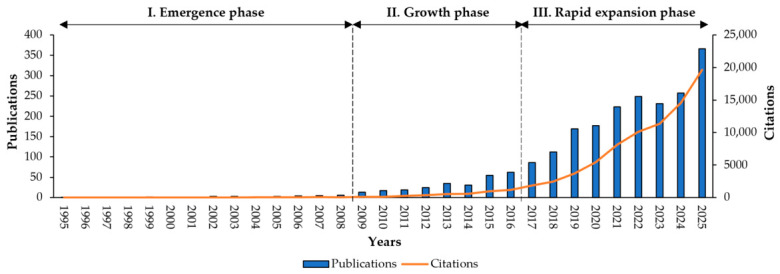
Annual evolution of publications and citations on the use of supercritical fluids for the extraction of bioactive compounds (1995–2025).

The evolution of scientific publications on the extraction of bioactive compounds using supercritical fluids during the period 1995–2025 is presented in Figure 1, based on a dataset comprising 2159 documents. To facilitate a clearer interpretation of the development of this research field, the publication timeline was divided into three distinct phases according to the observed trends in annual scientific output.

The first phase, defined as the emergence phase (1995–2008), was characterized by very low research productivity and a relatively stable publication trend, with only a few articles published per year. During this period, research on the application of supercritical fluids for the extraction of bioactive compounds was still at an early stage and had not yet attracted widespread scientific attention. Publications from this phase represent only 1.48% of the total documents identified in the dataset.

The second phase, referred to as the growth phase (2009–2016), exhibited a gradual and consistent increase in the number of publications. This period reflects the progressive development and consolidation of research on green extraction technologies, particularly the use of supercritical fluids for recovering high-value bioactive compounds from natural sources. Scientific output during this phase accounted for 11.90% of the total publications.

Finally, the third phase, termed the rapid expansion phase (2017–2025), showed a sharp increase in scientific production. During this stage, publication output grew significantly, representing 86.61% of the total documents included in the dataset. In parallel, citation counts also increased markedly, highlighting the growing scientific impact and the increasing relevance of this research area in the context of sustainable extraction technologies.

Table 1 provides a detailed comparison of the main bibliometric indicators across the three identified phases. The results reveal a sustained expansion of research on supercritical fluid extraction of bioactive compounds, reflected in the progressive increase in publication titles, documents, total citations, and authors, demonstrating the growing consolidation and scientific relevance of the field.

The second research phase (2009–2016) exhibited the highest average citations per document (82.08), largely driven by the publication of highly influential studies, including the most cited review article in the dataset [24]. In contrast, although the third phase (2017–2025) accumulated the highest total number of citations, the average citations per document decreased to 31.47. This indicator was calculated using the cumulative citations received up to the end of 2025 by publications from each research phase, divided by the number of documents in that phase. Therefore, the lower average observed in the most recent phase should not be interpreted as reduced scientific impact, but rather as a consequence of the shorter citation window available for recently published articles, which have had less time to accumulate citations.

A similar trend was observed for the main document types, particularly research articles, review articles, and proceedings papers. It should be noted that some publications are assigned to more than one document type in the Web of Science database (e.g., article and proceedings paper), resulting in overlapping classifications. Given this sustained growth in scientific production, it is important to further examine the main sources of this research output, including the most productive journals, authors, institutions, countries, and funding agencies, which are discussed in the following sections.

#### 2.1.2. Most Productive Journals

The most productive journals publishing research on supercritical fluid extraction of bioactive compounds are presented in Table 2. *Journal of Supercritical Fluids* ranks first with 201 publications (9.31% of the total output), confirming its central role in disseminating research on supercritical fluid technologies, including extraction processes, thermodynamics, and industrial applications. In contrast, journals such as *Food Chemistry* and *Molecules* are more oriented toward the analytical characterization and chemical properties of bioactive compounds, while *Industrial Crops and Products* emphasizes the valorization of plant resources and agro-industrial materials. Similarly, *Foods* and *Applied Sciences-Basel* provide a broader multidisciplinary platform for applied research in food systems and processing technologies. Review-oriented journals such as *Trends in Food Science & Technology* and *Critical Reviews in Food Science and Nutrition* tend to publish integrative and high-level analyses, which may explain their higher citation performance.

Several of the leading journals belong to the Q1 category, including *Food Chemistry*, *Industrial Crops and Products*, *Trends in Food Science & Technology*, *Antioxidants*, and *Critical Reviews in Food Science and Nutrition*, highlighting the high scientific quality and visibility of research in this field. In terms of impact, *Trends in Food Science & Technology* shows the highest impact factor (IF = 15.4) and the highest citations per publication (C/P = 112.43), indicating that review-based and broadly scoped journals tend to achieve higher average visibility. In contrast, *Journal of Supercritical Fluids* presents the highest h-index (H = 46), reflecting sustained and consistent scientific influence over time despite a lower average citation rate per article.

Journals such as *Food Chemistry* and *Critical Reviews in Food Science and Nutrition* also exhibit high citations per publication (51.71 and 56.31, respectively), reinforcing the relevance of journals with strong analytical and review-oriented scope. Meanwhile, *Molecules* and *Antioxidants* show intermediate citation performance, suggesting a balance between productivity and impact. Finally, the distribution of publishers reveals the strong presence of Elsevier and MDPI, which together account for the majority of the most productive journals in this research area.

#### 2.1.3. Most Productive Authors

Table 3 presents the ten most productive authors in the field of supercritical fluid extraction of bioactive compounds during the period 1995–2025. The results show that Maria Angela A. Meireles from the Universidade Estadual de Campinas is the most productive author with 45 publications, accumulating 1218 citations and an average of 27.07 citations per publication.

The second position is held by Elena Ibáñez from the Autonomous University of Madrid, with 38 publications and 2428 citations, highlighting a higher citation impact (63.89 citations per publication). Similarly, Julian Martinez, also affiliated with the Universidade Estadual de Campinas, ranks third with 34 publications and 1249 citations. Among the authors with the highest citation impact, Francisco J. Barba from the University of Valencia stands out with the highest average citation rate (100.58 citations per publication) and the highest H-index (94), indicating strong scientific influence within the field.

Overall, the results reveal a strong presence of researchers from Brazil and Spain, particularly from institutions such as the Universidade Estadual de Campinas and the Autonomous University of Madrid, which appear as key research hubs in the development of supercritical fluid technologies for the extraction of bioactive compounds.

#### 2.1.4. Most Productive Countries

**Figure 2:**
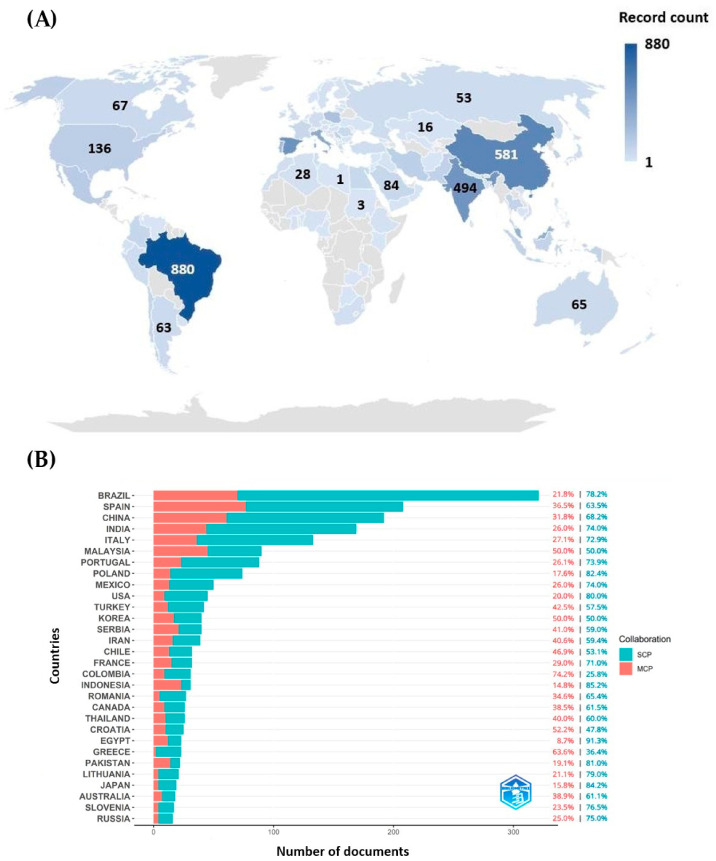
(**A**) Scientific production by country in research on supercritical fluid extraction of bioactive compounds (1995–2025). (**B**) Distribution of publications by corresponding author’s country, showing the number of single-country publications (SCPs) and multiple-country publications (MCPs) in research on supercritical fluid extraction of bioactive compounds (1995–2025).

The geographical distribution of scientific production is presented in Figure 2A. According to the Country Scientific Production indicator, Brazil recorded the highest number of author appearances in the dataset (880), followed by China (581) and Spain (504). These values represent the frequency with which authors affiliated with each country appear in the analyzed publications rather than the number of unique publications.

The predominance of Brazil and Spain is corroborated by the results presented in Table 3, where several of the most productive authors are affiliated with institutions from these countries. Notably, the most cited publications from both countries are review articles addressing the sustainable recovery of bioactive compounds from agro-industrial by-products (winery wastes) and food and natural products using intensified extraction technologies, including supercritical fluid extraction [36,37].

Figure 2B shows the distribution of publications according to the corresponding author’s country, distinguishing between single-country publications (SCPs) and multiple-country publications (MCPs). In this approach, each article is assigned to a single country based on the affiliation of the corresponding author, ensuring that the total frequency corresponds to the total number of publications analyzed.

Brazil remained the leading country in terms of corresponding author publications, followed by Spain, China, India, and Italy. Although Brazil contributed the largest number of publications, Spain and China exhibited higher levels of international collaboration, as reflected by their MCP percentages. Countries such as Indonesia, Pakistan, Egypt, Malaysia, and Serbia showed the highest proportions of MCPs, suggesting a greater dependence on international collaborations to advance research activities. Conversely, Poland, Romania, Greece, and Turkey were characterized by lower MCP percentages, indicating that their scientific production was predominantly developed through national collaborations.

The leading scientific production of Brazil, Spain, and China may reflect differences in research capacity, funding, natural resources, and national priorities. Brazil’s position is supported by its extensive agricultural biodiversity and sustained funding from CAPES, CNPq, and FAPESP, which have contributed to the consolidation of research hubs such as the Universidade Estadual de Campinas. In Spain, specialized institutions such as CSIC and CIAL, together with European funding frameworks and sustainability policies, including the European Green Deal, have promoted research on green extraction and agro-industrial by-product valorization. China’s rapid growth is consistent with increasing national investment in green technologies, natural products research, and sustainable manufacturing. Differences in SCP and MCP patterns also likely reflect variations in research capacity, funding opportunities, and access to specialized infrastructure. Brazil’s high proportion of single-country publications suggests a well-established domestic research network capable of sustaining scientific production independently, whereas Spain’s higher MCPs may be associated with collaborative European funding schemes. In contrast, the greater reliance on international collaborations observed in several emerging countries may facilitate access to specialized expertise, research infrastructure, and shared resources required for supercritical fluid extraction research.

### 2.2. Influential Publications and Leading Institutions

#### 2.2.1. Most Influential Articles

The most influential publications were identified based on the total number of citations. Table 4 lists the top 10 most cited articles related to the extraction of bioactive compounds between 1995 and 2025.

The most cited article is the review by Azmir et al. [24], with 1797 citations, which provides an overview of conventional and emerging techniques for extracting bioactive compounds from plant materials. Its comprehensive coverage of conventional and emerging extraction techniques, including supercritical fluid extraction, has made it a key reference in the field. It is followed by Xia et al. [38] with 788 citations, focusing on the biological activities and health benefits of grape polyphenols. The mentioned studies were published in the second phase (growth phase), while the following ones were published in the rapid expansion phase. The ones from the third phase include Lourenço et al. [39] and Ameer et al. [31], which discuss green extraction technologies for recovering polyphenols from plant matrices and agro-industrial by-products.

Several influential papers also address sustainable extraction technologies, such as Chemat et al. [36] and Barba et al. [37], highlighting environmentally friendly approaches for obtaining bioactive compounds. In addition, studies specifically focused on supercritical fluid extraction, including Pereira et al. [41] and da Silva et al. [22], have contributed to understanding the principles, applications, and economic aspects of this technology.

Beyond total citations, the C/Y indicator provides a time-normalized measure of citation impact by accounting for publication year. Although the review by Azmir et al. [24] was published in 2013, it remained the most influential publication in terms of both total citations and C/Y. In contrast, the high C/Y values of the more recent reviews by Lourenço et al. [39] and Chemat et al. [36] indicate their rapid scientific impact despite having had less time to accumulate citations.

#### 2.2.2. Leading Institutions

The most productive institutions in research on supercritical fluid extraction of bioactive compounds are presented in Table 5, considering total publications, total citations, citations per publication, and H-index.

The Universidade Estadual de Campinas (Brazil) ranks first with 124 publications, accumulating 3972 citations and an H-index of 38. The Consejo Superior de Investigaciones Científicas (CSIC, Spain) follows with 94 publications and the highest total citations (4 310), as well as the highest citations per publication (C/P = 45.85) among the leading institutions, indicating a strong scientific impact. The Instituto de Investigación en Ciencias de la Alimentación (CIAL-CSIC-UAM, Spain) ranks third with 75 publications, 2736 citations, and a C/P of 36.48.

Other notable institutions include the University of Novi Sad (Serbia) and several Brazilian universities, such as Universidade Federal de Santa Catarina, Universidade Federal de Santa Maria, and Universidade Estadual de Maringá. Similar to the trends observed for the most productive authors and countries, the leading affiliations are predominantly located in Brazil and Spain, highlighting the strong research activity in these countries in this field.

#### 2.2.3. Leading Funding Agencies

The main funding agencies supporting research in this field are presented in Table 6. As expected from the previous analyses of country productivity, Brazilian agencies show a clear predominance in research funding. In particular, Coordenação de Aperfeiçoamento de Pessoal de Nível Superior (CAPES) accounts for 10.65% of the total publications, followed by the Conselho Nacional de Desenvolvimento Científico e Tecnológico (CNPq) with 10.05%, and the Fundação de Amparo à Pesquisa do Estado de São Paulo (FAPESP) with 9.22%. This strong presence reflects the leading role of Brazil in scientific production related to supercritical fluid extraction of bioactive compounds. These agencies also present the highest h-index values in this analysis, indicating both high productivity and strong citation impact.

European funding bodies also play an important role, particularly the European Union (5.79%), the Spanish Government (5.19%), and the Fundação para a Ciência e a Tecnologia (FCT) from Portugal (3.75%), which is consistent with the high research activity previously observed in these countries. Additionally, the National Natural Science Foundation of China (2.50%) appears among the main funding agencies, reinforcing the contribution of Asian research institutions. Interestingly, the Consejo Nacional de Ciencia y Tecnología (CONACYT) from Mexico (1.11%) also appears among the top funding organizations and shows a relatively high citation impact (C/P = 42.88), placing it among the agencies with the highest citation performance despite its lower share of publications.

Regarding citation impact, some agencies exhibit particularly high citations per publication (C/P), especially the Fundação para a Ciência e a Tecnologia (56.35) and the Marie Curie Actions (54.25), suggesting that research supported by these programs tends to achieve a strong citation impact.

### 2.3. Research Trends and Thematic Structure

#### 2.3.1. Keyword Analysis: Word Cloud and Co-Occurrence Network

**Figure 3:**
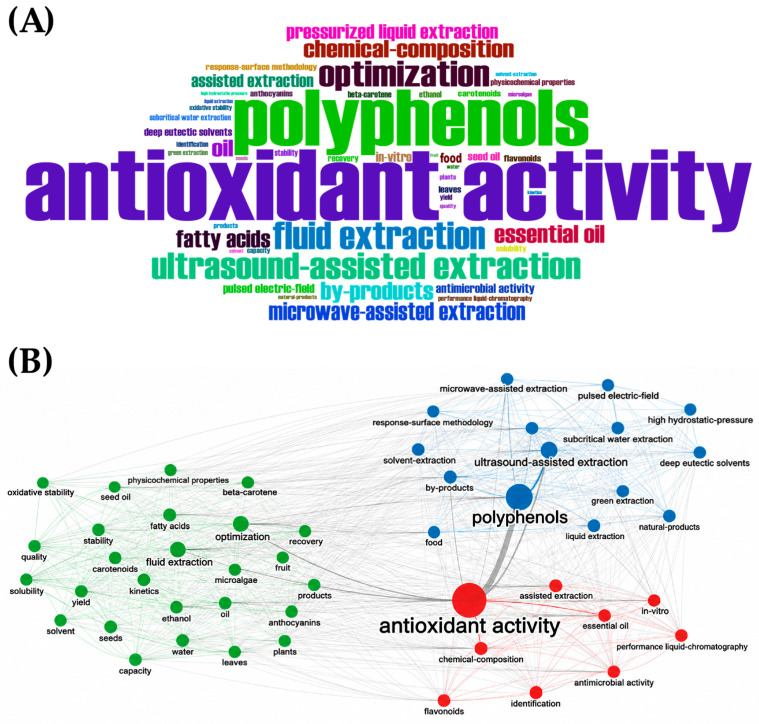
(**A**) Word cloud of the most relevant keywords in research on supercritical fluid extraction of bioactive compounds (1995–2025). (**B**) Keyword co-occurrence network of the most relevant terms in research on supercritical fluid extraction of bioactive compounds (1995–2025).

Keyword analysis was performed to identify the main research themes in the scientific literature on supercritical fluid extraction. In this study, the analysis was based on Keywords Plus, a set of terms automatically generated by the Web of Science database from the titles of cited references, which often reveal additional conceptual relationships within the scientific literature. The word cloud presented in Figure 3A illustrates the 50 most frequent terms identified in publications related to the extraction of bioactive compounds using supercritical fluids during the period 1995–2025, where the size of each term reflects its frequency of appearance. The most prominent keywords include “antioxidant activity” (816 occurrences) and “polyphenols” (623), followed by “fluid extraction” (321), “ultrasound-assisted extraction” (317), “optimization” (299), and “by-products” (213). These results indicate that research in this field is mainly focused on the recovery of antioxidant compounds, particularly polyphenols, as well as on improving extraction efficiency and the valorization of agro-industrial by-products.

In addition, several extraction techniques, such as “microwave-assisted extraction” (189), “pressurized liquid extraction” (159), “deep eutectic solvents” (88), and “subcritical water extraction” (67), appear with high frequency, reflecting either the integration of supercritical fluid extraction with other green extraction technologies [43] or comparative studies evaluating their performance [44,45,46]. Other relevant terms, including “fatty acids” (205), “essential oil” (204), “chemical composition”, “antimicrobial activity” (104), and “carotenoids” (80), indicate the diversity of compounds and applications investigated.

A keyword co-occurrence network was constructed to examine the relationships among the most relevant research topics in the field (Figure 3B). Network metrics, including betweenness centrality (which measures the extent to which a node lies on shortest paths between other nodes), closeness centrality (which reflects the average distance of a node to all other nodes in the network), and PageRank (which evaluates node importance based on its connections to other highly connected nodes), were used to assess keyword relevance [47].

The keyword co-occurrence network, consisting of 50 terms (nodes), reveals three main clusters. The red cluster is centered on “antioxidant activity”, which shows the highest PageRank (0.114) and betweenness centrality (160.205), indicating its dominant role as a core theme connecting different research areas within the context of supercritical fluid extraction. This cluster is associated with terms such as “essential oil”, “chemical composition”, “assisted extraction”, and “antimicrobial activity”, reflecting a strong focus on the characterization and biological properties of bioactive compounds obtained using supercritical fluids.

The blue cluster is structured around “polyphenols” (PageRank = 0.088), which also exhibits high betweenness centrality (90.297), highlighting its importance as a key research topic. This cluster is closely linked to extraction techniques such as “ultrasound-assisted extraction”, “microwave-assisted extraction”, “pressurized liquid extraction”, “pulsed electric field”, and “subcritical water extraction”, indicating that supercritical fluid extraction is frequently integrated with or compared to other green and intensified extraction technologies. The presence of “by-products” reflects the growing interest in agro-industrial residues as sustainable sources of bioactive compounds, while that of “deep eutectic solvents” further suggests the exploration of alternative solvent systems in combination with supercritical processes.

The green cluster is centered on “optimization” (PageRank = 0.046), which acts as a transversal theme associated with process variables and extraction performance in supercritical fluid extraction, including “fatty acids”, “carotenoids”, “recovery”, “solubility”, and “ethanol”. The presence of “ethanol” within this cluster suggests the use of co-solvents in supercritical CO_2_ extraction to enhance the recovery of moderately polar compounds [22], linking process optimization with solvent selection strategies.

The co-occurrence of “ethanol” and “deep eutectic solvents” also highlights future research opportunities in co-solvent development. Although ethanol remains the predominant co-solvent in supercritical CO_2_ extraction, the increasing interest in deep eutectic solvents suggests their potential as more sustainable alternatives. Future studies should also incorporate techno-economic analyses and life cycle assessment (LCA) to evaluate the economic feasibility and environmental impacts associated with industrial-scale implementation of these extraction processes.

Another important challenge is the limited availability of pilot- and industrial-scale studies. Future research should focus on continuous processing, scale-up strategies, and process validation under industrial operating conditions to facilitate the commercial implementation of supercritical fluid extraction technologies.

Overall, the co-occurrence network reveals a progressive evolution of research from bioactive compound characterization to process optimization and sustainable extraction strategies, highlighting the increasing maturity of the field and the opportunities for further technological development.

#### 2.3.2. Thematic Evolution

**Figure 4:**
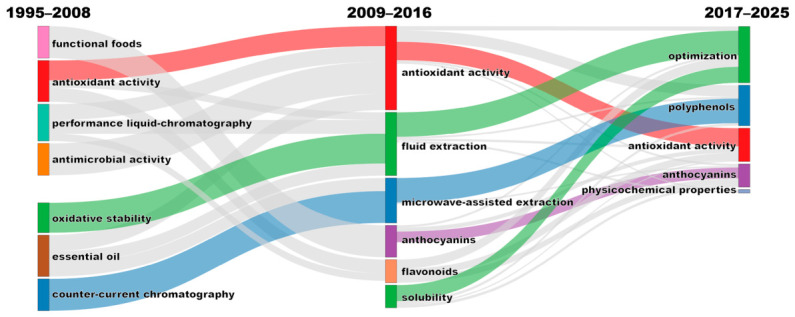
Thematic evolution of research on supercritical fluid extraction of bioactive compounds (1995–2025).

The thematic evolution of research on supercritical fluid extraction of bioactive compounds across the three defined periods is illustrated in Figure 4. During the first period (1995–2008), research focused primarily on compound characterization and analytical methods. Between 2009 and 2016, the field expanded toward specific classes of bioactive compounds, particularly anthocyanins and flavonoids, together with complementary green extraction technologies such as microwave-assisted extraction. In the most recent period (2017–2025), optimization emerged as the dominant research theme, accompanied by increasing interest in polyphenols, physicochemical properties, and process-oriented studies, reflecting the growing specialization of the field.

**Figure 5:**
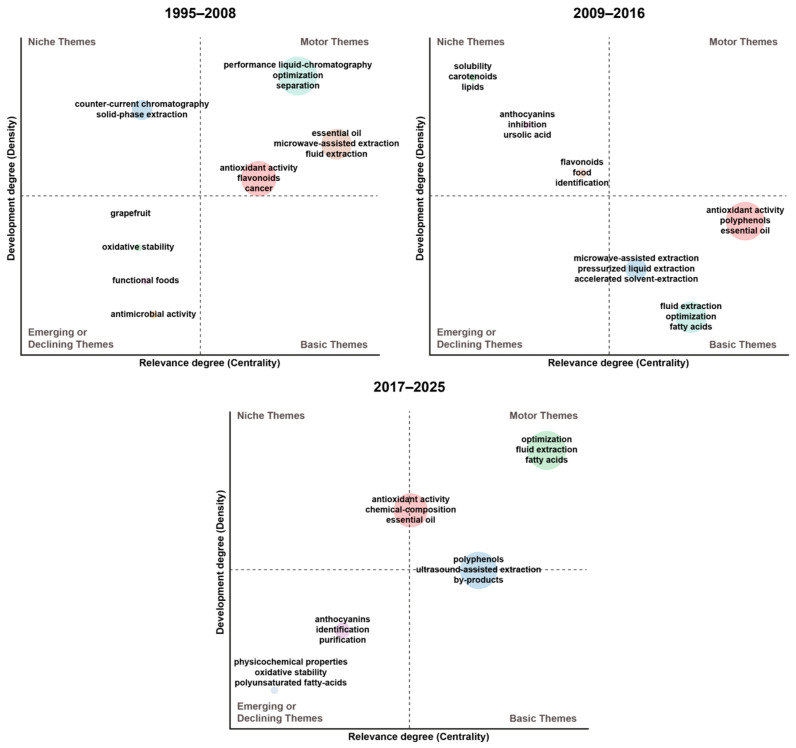
Thematic maps of research on supercritical fluid extraction of bioactive compounds across three periods.

To provide a more detailed understanding of the conceptual structure of the research field, thematic maps were analyzed for each period (Figure 5). These maps classify research themes according to their centrality (relevance within the field) and density (degree of internal development), allowing their distribution into four categories: motor themes, basic themes, niche themes, and emerging or declining themes.

In the first period (1995–2008), a total of eight thematic clusters were identified. Motor themes are dominated by performance liquid chromatography, optimization, and separation, which show the largest bubble sizes, highlighting their dominant role in early research. Other motor themes include essential oil, microwave-assisted extraction, and fluid extraction, reflecting an early emphasis on green extraction technologies, as well as antioxidant activity, flavonoids, and cancer, indicating a strong focus on the characterization and biological evaluation of bioactive compounds. In contrast, antimicrobial activity, functional foods, oxidative stability, and grapefruit appear as emerging or declining themes with relatively small bubble sizes, whereas counter-current chromatography and solid-phase extraction correspond to niche themes with moderate development but limited interaction with other research topics.

In the second period (2009–2016), six thematic clusters were identified. Antioxidant activity became a basic theme, reflecting its continued relevance as a central research topic in the field, while polyphenols and essential oil also emerged as basic themes, reflecting the growing interest in specific groups of bioactive compounds. Optimization likewise emerged as a basic theme, highlighting the growing interest in optimizing process parameters to maximize extraction yield and efficiency. In addition, other green extraction technologies, including microwave-assisted extraction, pressurized liquid extraction, and accelerated solvent extraction, gained relevance as basic themes, reflecting the growing interest in alternative extraction approaches alongside supercritical fluid extraction. Niche themes included specific groups of bioactive compounds, such as carotenoids, anthocyanins, and flavonoids, together with topics related to food, identification, lipids, inhibition, and ursolic acid, representing more specialized research directions.

In the most recent period (2017–2025), five thematic clusters were identified. Motor themes were dominated by optimization, fluid extraction, and fatty acids, highlighting the consolidation of process optimization as a central focus of supercritical fluid extraction research. Antioxidant activity, chemical composition, and essential oil were positioned between the niche and motor themes, reflecting their continued importance in the characterization of extracts and the evaluation of their biological properties. Polyphenols, ultrasound-assisted extraction, and by-products were located between the basic and motor themes, reflecting the increasing integration of ultrasound with supercritical fluid extraction, either as a pretreatment [43,48] or as a complementary extraction step after supercritical fluid extraction [49], to improve the recovery of bioactive compounds from plant matrices and agro-industrial by-products. The remaining clusters were classified as emerging or declining themes, representing less consolidated research directions within the field.

The observed thematic evolution reflects the progressive maturation of supercritical fluid extraction research. Early studies were primarily focused on demonstrating the feasibility of the technology and characterizing extracted bioactive compounds, whereas subsequent research progressively shifted toward process optimization, hybrid extraction strategies, and the valorization of agro-industrial by-products. This transition is also consistent with efforts to overcome the limited ability of pure supercritical CO_2_ to efficiently recover polar bioactive compounds, promoting the development of co-solvent systems and the integration of complementary extraction technologies. These developments also reflect the increasing demand for more efficient, sustainable, and scalable extraction processes across the food, nutraceutical, and pharmaceutical industries.

#### 2.3.3. Citation Topics Analysis (Meso- and Micro-Level)

To further explore the thematic structure of the scientific literature, a citation topic analysis was conducted using the hierarchical classification system provided by Web of Science. This approach groups publications into thematic areas based on citation relationships, allowing the identification of broader research domains (meso-level topics) as well as more specific research niches (micro-level topics).

The meso-level citation topics provide an overview of the main thematic areas in which the analyzed publications are concentrated. As shown in Table 7, the majority of the publications are associated with the topic phytochemicals, which represents 45.72% of the records. This result highlights the strong focus of the research field on the extraction and characterization of plant-derived bioactive compounds. The second most relevant meso-level topic is Ionic, Molecular and Complex Liquids (19.82%), which reflects the importance of solvent systems and advanced extraction media in studies involving supercritical fluid technologies. Other notable thematic areas include lipids (8.89%) and Photoproductivity (7.13%), indicating research interest in lipid-rich matrices, natural pigments, and other biologically relevant compounds obtained from different biological sources.

A more detailed perspective of the research landscape is provided by the micro-level citation topics, which represent specific research niches within the broader thematic areas. As shown in Table 7, the most prominent topic is antioxidant activity, accounting for 31.96% of the publications. This finding indicates that a substantial proportion of the research is focused on evaluating the bioactivity of compounds obtained through supercritical fluid extraction. The topic supercritical carbon dioxide (18.43%) also represents a major research focus, reflecting the widespread use of CO_2_ as the primary solvent in supercritical extraction processes. Other relevant topics include Virgin Olive Oil (8.43%) and essential oil (4.40%), highlighting the importance of lipid-rich matrices and aromatic compounds as target products in extraction studies.

In addition, several micro-level topics reveal growing interest in specific high-value compounds and natural products, including carotenoids, bioactive polysaccharides, functional bioactive peptides, and stimulant compounds such as caffeine. Other topics related to natural bioactive substances, such as cannabinoids, propolis, curcumin, and thymoquinone, further illustrate the diversity of compounds investigated using supercritical fluid extraction technologies. Additionally, the presence of topics related to ionic liquids highlights increasing research attention toward the development of alternative solvent systems and green extraction media aimed at improving the efficiency and selectivity of bioactive compound recovery.

#### 2.3.4. Alignment with the United Nations Sustainable Development Goals (SDGs)

The United Nations 2030 Agenda for Sustainable Development established 17 Sustainable Development Goals (SDGs) aimed at addressing global challenges related to health, environmental protection, sustainable resource use, and economic development. In the Web of Science database, the relationship between scientific publications and the SDGs is determined through a mapping between SDGs and Micro Citation Topics, where each SDG is linked to a set of micro-level research topics. Publications associated with these topics are then linked to the corresponding SDG defined in the Web of Science mapping, allowing the evaluation of how different research fields contribute to global sustainability priorities. Accordingly, because this classification is based on predefined Micro Citation Topics rather than the publication date, publications from the entire study period (1995–2025) were included in the SDG analysis, even though the SDGs were formally adopted in 2015.

**Figure 6:**
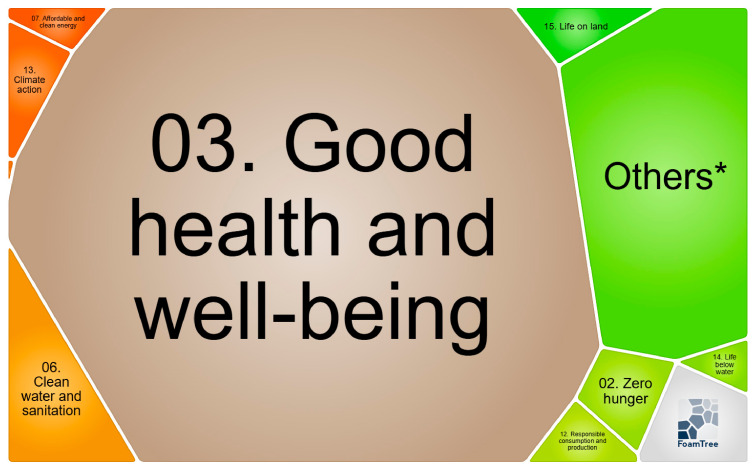
Distribution of publications on supercritical fluid extraction of bioactive compounds across the United Nations Sustainable Development Goals (SDGs) (1995–2025). * Others represents documents without an SDG classification in the Web of Science database.

The distribution of publications according to the SDGs is presented in Figure 6. The results show that most of the research in this field is strongly associated with SDG 3 (Good Health and Well-Being), representing 70.96% of the publications. This predominance is consistent with the Micro Citation Topics identified in the previous analysis, many of which are mapped to SDG 3, including topics related to antioxidant activity, essential oils, carotenoids, polysaccharide bioactivities, functional bioactive peptides, coffee and caffeine, cannabinoids, propolis, curcumin, thymoquinone, flavonoids, and other phytochemical compounds with potential health-related applications.

Other SDGs appear with lower representation, including SDG 6 (Clean Water and Sanitation), with 4.49% of the publications, SDG 2 (Zero Hunger), with 1.85%, and SDG 13 (Climate Action), with 1.34%. Smaller contributions were also observed for SDG 12 (Responsible Consumption and Production), SDG 7 (Affordable and Clean Energy), SDG 15 (Life on Land), and SDG 14 (Life Below Water), according to the Web of Science SDG mapping. Overall, these results indicate that studies on supercritical fluid extraction are primarily associated with health-related research areas while also showing more limited connections with other sustainability goals defined in the 2030 Agenda.

## 3. Methodology

**Figure 7:**
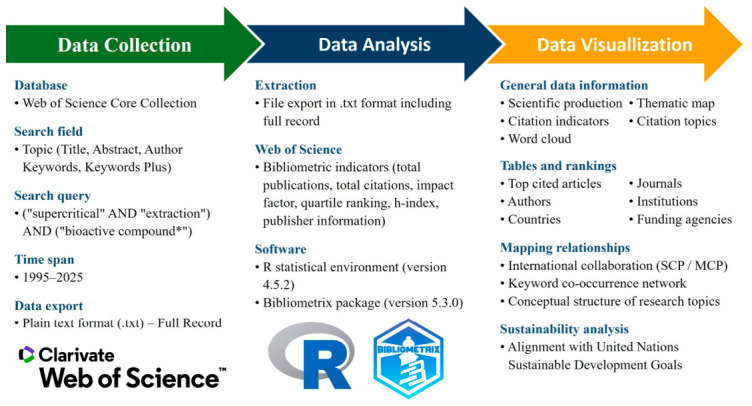
Bibliometric analysis workflow. *: Wildcard operator.

The overall workflow of the bibliometric analysis conducted in this study is presented in Figure 7, which summarizes the main stages, including data collection, data analysis, and visualization of the bibliometric results.

### 3.1. Data Collection

A bibliometric dataset was retrieved from the Web of Science Core Collection (WoSCC) database. The search was performed using the following query:

(“supercritical” AND “extraction”) AND (“bioactive compound*”).

The search was conducted in the Topic field, which includes title, abstract, author keywords, and Keywords Plus. The time span was restricted to publications between January 1995 and December 2025 in order to analyze the evolution of research on supercritical fluid extraction of bioactive compounds over the last three decades.

All records were exported from WoSCC as Plain Text files (.txt) using the Full Record option. The exported dataset included a wide range of bibliographic information, such as authors, titles, abstracts, keywords, journals, institutional affiliations, countries, document types, and citation indicators, among other metadata available in the Web of Science database. The complete bibliometric dataset analyzed in this study is provided in the Supplementary Materials.

### 3.2. Data Analysis

Several bibliometric indicators were obtained directly from the WoSCC database, which provides analytical tools for evaluating publication and citation metrics. These indicators included total publications, total citing articles, journal impact factor, quartile ranking, h-index, and publisher information. This information was used to evaluate the scientific productivity and impact of journals and publication sources within the research field.

In addition to the indicators provided by the Web of Science database, further bibliometric analyses were performed using the Bibliometrix package (version 5.3.0) in the R statistical environment (version 4.5.2). After downloading the records from WoSCC in plain text format (.txt), R was opened, and the Bibliometrix package was loaded. Subsequently, the biblioshiny() function was executed to launch the Biblioshiny interface.

Within the Biblioshiny interface, the dataset was imported through the Data section by selecting Import or Load and then Import raw file(s), where the WoS plain text file was uploaded. This procedure enabled the import and organization of the bibliographic records in a format suitable for bibliometric analysis within the Bibliometrix environment.

To improve the consistency and interpretability of the keyword and thematic evolution analyses, a data cleaning procedure was applied in the Biblioshiny environment using the functions “Load a list of synonyms” and “Load a list of terms to remove”. In the synonym normalization step, different variants referring to the same concept were grouped under a unified term. Specifically, the following synonym pairs were defined: “supercritical fluid extraction” with “supercritical-fluid extraction”, “supercritical carbon-dioxide”, “supercritical extraction”, “carbon-dioxide extraction”, “co2 extraction”, “supercritical co2”, “co2”, “carbon-dioxide”, “supercritical co2 extraction”, “supercritical carbon dioxide”, and “supercritical carbon dioxide”; “polyphenols” with “phenolic compounds” and “phenolic-compounds”; “antioxidant activity” with “antioxidant activities”, “antioxidant capacity”, “antioxidant”, “antioxidant properties”, and “antioxidants”; “ultrasound-assisted extraction” with “ultrasound”; “pulsed electric field” with “pulsed electric fields”; “essential oil” with “essential oils”; “fatty acids” with “fatty-acid-composition”, “fatty-acids”, and “acid”; and “antimicrobial activity” with “antibacterial activity”. Additionally, a list of non-informative or overly generic terms was removed using the “Load a list of terms to remove” function. The excluded terms were “supercritical fluid extraction”, “bioactive compounds”, “extraction”, “extraction techniques”, “l”, and “l.”, as these terms are either inherent to the search query or do not contribute to distinguishing thematic patterns.

### 3.3. Data Visualization

The bibliometric results were organized into four main analytical components to facilitate the interpretation of the scientific landscape of research on supercritical fluid extraction of bioactive compounds: overview of the scientific production, scientific production and productivity, influential publications and leading institutions, and research trends and thematic structure.

Several descriptive indicators and graphical representations, including the overview of scientific production, annual publication and citation trends, most productive journals, authors, institutions, influential articles, funding agencies, and citation topics at both meso and micro levels, were obtained directly from the analytical tools available in the WoSCC database. The geographical distribution of scientific production by country was also derived from WoSCC data, and a corresponding map was generated in Microsoft Excel. Additionally, the alignment of publications with the United Nations Sustainable Development Goals (SDGs) was visualized using FoamTree 3.6.1 (https://get.carrotsearch.com/, accessed on 10 June 2026), based on the SDG mapping provided by the Web of Science database.

Collaboration patterns among countries, including single-country publications (SCPs) and multiple-country publications (MCPs), and keyword-based analyses, such as word cloud visualizations and keyword co-occurrence networks, were analyzed and visualized using the Bibliometrix 5.3.0 package through the Biblioshiny interface.

## 4. Conclusions

The bibliometric analysis of research on the extraction of bioactive compounds using supercritical fluids reveals a sustained and accelerated growth in scientific production since the mid-1990s, with a particularly strong expansion during the last decade. This trend reflects the increasing interest in environmentally friendly extraction technologies and the growing demand for natural bioactive compounds with applications in the food and nutraceutical industries.

The results identify Brazil as the leading contributor to this research field, followed by China and Spain. This leadership is reflected in the presence of the most productive and highly cited authors, as well as several of the leading research institutions and funding agencies identified in the analysis. The evaluation of collaboration patterns also indicates the presence of international research partnerships among the most productive countries, contributing to the dissemination and development of research on supercritical fluid extraction.

The thematic analysis of keywords, thematic evolution, and thematic mapping indicates that the literature has progressively shifted from compound characterization to process optimization and application-oriented research. While topics such as antioxidant activity and polyphenols remain central, increasing attention has been given to process efficiency, assisted extraction technologies, and the recovery of specific compounds such as anthocyanins, flavonoids, fatty acids, and essential oils. Additionally, the growing interest in the valorization of agro-industrial by-products reflects a broader trend toward sustainable resource utilization. The alignment of the identified research topics with the United Nations Sustainable Development Goals, particularly SDG 3 (Good Health and Well-Being), further highlights the relevance of this research area in the context of sustainable development.

Importantly, the findings of this study highlight the potential of supercritical fluid extraction as a key technology for the sustainable production of high-value food ingredients, supporting innovation in food processing and the development of functional foods. However, translating these advances into industrial applications will require further research on process scalability, techno-economic feasibility, and environmental sustainability. Future studies should also explore hybrid extraction processes and sustainable co-solvent systems to improve the recovery of polar bioactive compounds while facilitating the wider adoption of supercritical fluid extraction technologies.

Despite providing valuable insights into the evolution and structure of research in this field, certain limitations should be considered. The analysis was restricted to publications indexed in the Web of Science Core Collection database and focused exclusively on scientific literature, without considering other sources of technological development such as patents, technical reports, or industrial documentation. Future studies integrating multiple databases and additional document types could provide a more comprehensive perspective on the scientific and technological landscape of supercritical fluid extraction.

## Figures and Tables

**Table 1:** Bibliometric indicators by research phase (1995–2025).

Category	Indicator	1995–2008	2009–2016	2017–2025
Main Information About the Data	Publication Titles	29	118	380
	Documents	32	257	1870
	Total Citations (1995–2025)	1708	21,094	58,852
	Average Citations per Doc	53.38	82.08	31.47
	Authors	92	973	7390
Document Types	Article	21	180	1190
	Review Article	8	66	672
	Proceeding Paper	2	14	24
	Early Access	-	-	15
	Meeting Abstract	1	5	-
	Book Chapters	-	1	2
	Data Paper	-	-	1
	Letter	1	-	-

**Table 2:** Top 10 most productive journals in research on the supercritical fluid extraction of bioactive compounds and their bibliometric indicators (1995–2025).

Rank	Journals	TP	TC	C/P	IF	Q	H	Publisher
1	*Journal of Supercritical Fluids*	201	4095	20.37	4.4	2	46	Elsevier
2	*Molecules*	129	5343	41.42	4.6	2	39	MDPI
3	*Foods*	96	3067	31.95	5.1	1	31	MDPI
4	*Food Chemistry*	63	3258	51.71	9.8	1	28	Elsevier
5	*Industrial Crops and Products*	44	1717	39.02	6.2	1	25	Elsevier
6	*Trends in Food Science Technology*	44	4947	112.43	15.4	1	38	Elsevier
7	*Processes*	39	1064	27.28	2.8	3	18	MDPI
8	*Antioxidants*	37	1423	38.46	6.6	1	19	MDPI
9	*Applied Sciences-Basel*	37	578	15.62	2.5	2	14	MDPI
10	*Critical Reviews in Food Science and Nutrition*	36	2027	56.31	8.8	1	23	Taylor & Francis

TP: total publications; TC: Total Cites; C/P: cites per publication; IF: Impact factor; Q: Quartile; H: h-index.

**Table 3:** Top 10 most productive authors in research on supercritical fluid extraction of bioactive compounds and their bibliometric indicators (1995–2025).

Rank	Author	Affiliation	Country	TP	TC	C/P	H
1	Meireles, Maria Angela A.	Universidade Estadual de Campinas	Brazil	45	1218	27.07	59
2	Ibáñez, Elena	Autonomous University of Madrid	Spain	38	2428	63.89	76
3	Martinez, Julian	Universidade Estadual de Campinas	Brazil	34	1249	36.74	44
4	Putra, Nicky	Higher Colleges of Technology	United Arab Emirates	33	438	13.27	25
5	Cardozo-Filho, Lucio	Universidade Estadual de Maringa	Brazil	28	507	18.11	41
6	Jokic, Stela	University of JJ Strossmayer Osijek	Croatia	27	830	30.74	38
7	Cifuentes, Alejandro	Autonomous University of Madrid	Spain	26	1477	56.81	70
8	Barba, Francisco J.	University of Valencia	Spain	26	2615	100.58	94
9	Zabot, Giovani Leone	Universidade Federal de Santa Maria	Brazil	25	442	17.68	34
10	Ferreira, Sandra R. S.	Universidade Federal de Santa Catarina	Brazil	25	424	16.96	30

TP: total publications; TC: Total Cites; C/P: cites per publication; H: h-index.

**Table 4:** Most cited publications in research on the extraction of bioactive compounds using supercritical fluids (1995–2025).

Rank	Article Title	Year	Type	TC	C/Y	Reference
1	Techniques for extraction of bioactive compounds from plant materials: A review	2013	R	1797	128.4	[24]
2	Biological Activities of Polyphenols from Grapes	2010	R	788	46.4	[38]
3	Antioxidants of Natural Plant Origins: From Sources to Food Industry Applications	2019	R	725	90.6	[39]
4	Green Extraction Methods for Polyphenols from Plant Matrices and Their Byproducts: A Review	2017	R	590	59.0	[31]
5	Essential Oils: Extraction, Bioactivities, and Their Uses for Food Preservation	2014	A	574	44.2	[40]
6	A review of sustainable and intensified techniques for extraction of food and natural products	2020	R	512	73.1	[36]
7	Green alternative methods for the extraction of antioxidant bioactive compounds from winery wastes and by-products: A review	2016	R	484	44.0	[37]
8	Supercritical Fluid Extraction of Bioactive Compounds: Fundamentals, Applications and Economic Perspectives	2010	R	467	27.5	[41]
9	Enzyme assisted extraction of biomolecules as an approach to novel extraction technology: A review	2018	R	460	51.1	[42]
10	Supercritical fluid extraction of bioactive compounds	2016	R	459	41.70	[22]

TC: Total Cites; C/Y: cites per year; R: review; A: article.

**Table 5:** Leading institutions and their bibliometric indicators in research on supercritical fluid extraction of bioactive compounds (1995–2025).

Rank	Affiliations	Country	TP	TC	C/P	H
1	Universidade Estadual De Campinas	Brazil	124	3972	32.03	38
2	Consejo Superior de Investigaciones Científicas (CSIC)	Spain	94	4310	45.85	35
3	CSIC UAM Instituto de Investigación en Ciencias de la Alimentación (CIAL)	Spain	75	2736	36.48	31
4	University of Novi Sad	Serbia	46	1094	23.78	19
5	Universidade Federal de Santa Catarina (UFSC)	Brazil	44	996	22.64	20
6	Egyptian Knowledge Bank (EKB)	Egypt	37	1315	35.54	14
7	Universidade Federal de Santa Maria (UFSM)	Brazil	36	912	25.33	18
8	Universiti Teknologi Malaysia	Malaysia	35	1070	30.57	19
9	Universidad de Cadiz	Spain	32	1079	33.72	14
10	Universidade Estadual de Maringa	Brazil	32	564	17.63	16

TP: total publications; TC: Total Cites; C/P: cites per publication; H: h-index.

**Table 6:** Main funding agencies supporting research on supercritical fluid extraction of bioactive compounds (1995–2025).

Rank	Funding Agency	Country/Region	TP	TC	C/P	H
1	Coordenação de Aperfeiçoamento de Pessoal de Nível Superior (CAPES)	Brazil	230	5181	22.53	43
2	Conselho Nacional de Desenvolvimento Científico e Tecnológico (CNPq)	Brazil	217	4824	22.23	42
3	Fundação de Amparo à Pesquisa do Estado de São Paulo (FAPESP)	Brazil	199	4810	24.17	43
4	European Union (EU)	European Union	125	4536	36.29	39
5	Spanish Government	Spain	112	4647	41.49	37
6	Fundação para a Ciência e a Tecnologia (FCT)	Portugal	81	4564	56.35	37
7	National Natural Science Foundation of China (NSFC)	China	54	1194	22.11	22
8	Consejo Nacional de Ciencia y Tecnología (CONACYT)	Mexico	24	1029	42.88	17
9	Marie Curie Actions	European Union	24	1302	54.25	15
10	Ministry of Education, Science & Technological Development, Serbia	Serbia	21	394	18.76	12

TP: total publications; TC: Total Cites; C/P: cites per publication; H: h-index.

**Table 7:** Meso- and micro-level citation topics identified in research on supercritical fluid extraction of bioactive compounds (1995–2025).

Topic ID	Topic	Record Count	% *
Meso			
3.16	Phytochemicals	987	45.72
2.89	Ionic, Molecular and Complex Liquids	428	19.82
1.68	Lipids	192	8.89
3.171	Photoproductivity	154	7.13
3.85	Food Science and Technology	106	4.91
1.287	Dietary Stimulants	27	1.25
1.100	Substance Abuse	24	1.11
3.87	Paper and Wood Materials Science	17	0.79
3.4	Crop Science	16	0.74
1.141	Hormone Therapy	13	0.60
2.166	Chromatography and Electrophoresis	13	0.60
1.79	Molecular and Cell Biology—Physiology	12	0.56
3.2	Marine Biology	12	0.56
3.220	Smell and Taste Science	11	0.51
3.32	Entomology	11	0.51
Micro			
3.16.28	Antioxidant Activity	690	31.96
2.89.1121	Supercritical Carbon Dioxide	398	18.43
1.68.621	Virgin Olive Oil	182	8.43
3.16.314	Essential Oil	95	4.40
3.171.477	Microalgae Biotechnology	90	4.17
3.171.1011	Carotenoids	64	2.96
3.16.698	Polysaccharide Bioactivities	42	1.95
3.85.1687	Functional Bioactive Peptides	33	1.53
1.287.1516	Coffee And Caffeine	26	1.20
2.89.508	Ionic Liquids	25	1.16
1.100.625	Cannabinoids	24	1.11
3.16.1671	Propolis	22	1.02
3.16.1399	Curcumin	20	0.93
3.85.554	Advanced Food Drying	19	0.88
3.16.2237	Thymoquinone	17	0.79

* Percentage calculated based on the total number of publications (2159).

## Data Availability

The data supporting the findings of this study are available in the Supplementary Materials.

## Supplementary Materials

The following supporting information can be downloaded at: https://www.mdpi.com/article/10.3390/molecules31142511/s1.

## Abbreviations

The following abbreviations are used in this manuscript:

C/P	Citations per publication
CAPES	Coordenação de Aperfeiçoamento de Pessoal de Nível Superior
CIAL	Instituto de Investigación en Ciencias de la Alimentación
CNPq	Conselho Nacional de Desenvolvimento Científico e Tecnológico
CO_2_	Carbon dioxide
CONACYT	Consejo Nacional de Ciencia y Tecnología
CSIC	Consejo Superior de Investigaciones Científicas
EU	European Union
FAPESP	Fundação de Amparo à Pesquisa do Estado de São Paulo
FCT	Fundação para a Ciência e a Tecnologia
H	h-index
IF	Impact factor
MCPs	Multiple-country publications
NSFC	National Natural Science Foundation of China
Q	Quartile
R	Review article
SCPs	Single-country publications
SDGs	Sustainable Development Goals
scCO_2_	Supercritical carbon dioxide
TC	Total Cites
TP	Total publications
UFSC	Universidade Federal de Santa Catarina
UFSM	Universidade Federal de Santa Maria
WoSCC	Web of Science Core Collection
